# Legal Recognition and Protection

**Published:** 1867-11

**Authors:** 


### Editorial.

### LEGAL RECOGNITION AND PROTECTION.

As most of our readers are aware, a movement in this direction has been on foot for two years past; but without a definite result so far as obtaining the object sought was concerned, and still not without very considerable results in the way of agitation, and awaking attention to the matter. The movement was first made in Ohio; and since that time it has been taken up by almost all the neighboring States, and in some of them the prospect of an early success seems far stronger than in our own; though the presumption is very strong that such a law would have been in full operation in this State at this time, had proper attention been given by the profession to the matter at the last session of the Legislature. At that time there seemed to be nobody to give it needed attention. If the matter is ever accomplished it must be by the active efforts of the members of the Dental profession, and that too by all who feel an interest in it.

It is useless, and wrong for a work of such great and general interest to be devolved on the shoulders of three or four persons. There should be a hearty co-operation upon the part of every member of the profession in the State, who feels an interest in it.

The necessity of a law for the protection, primarily, of the people from the hands of the empiric and unqualified; and secondarily, for the protection of the true Dental profession from the evil influences accruing from the same source, is so apparent as scarcely to require mention.

In regard to the first, let the many thousands of cases of irreparable mutilation, and of intense and protracted suffering, in many instances bringing the sufferers to an untimely death, testify. Oh that such cases could speak with trumpet tongues, to ring through all the land the wrongs and outrages committed! Our legislators would then hasten as one man to grant the needed law. We are almost daily witnesses of mal-practice by those professing to be Dentists, that ought to consign the perpetrators to a prison, if not a worse fate. Perhaps this may be regarded as an extreme view. Could we bring the witnesses upon the stand, we could prove it beyond all possibility of a cavil to be a mild view. What! Shall men, women and tender children be marred, mutilated and rendered miserable for life, by having the beautiful, symmetrical human face, through which the soul shines and speaks transformed into a deformed and unsightly thing, through which the soul refuses to give its correct utterance; and no one raise a warning voice, and none to ask for that protection which they should have? Nay, verily!

Hundreds and perhaps thousands of these incompetents are prowling through this and the immediate neighboring States, committing their depredations wherever an opportunity offers, without let or hindrance. But it is said, that "the people love to be humbugged." It is not true; it is a libel on humanity, and generally those who make such utterance know it. People many a time submit to wrongs and grievously injurious things, and it may be at the time very willingly too; but always to repent it when true knowledge comes. People submit to these things because of a want of knowledge on their part, and through the falsehood, deceit and misrepresentation of others. Knowledge on the part of the people would remedy this, as well as many other evils; but that will be the work of years, and perhaps of generations, and to meet the difficulty in the meantime something else must be done, and we can conceive of nothing better than stringent legislation. It has been said that the proper education of the Dentist would remedy the difficulty. This would only accomplish the object when all men become honest, and when every one who chooses to enter the ranks of the profession, can be trained to the possession of the requisite ability to perform its responsible duties. Experience teaches us that many seek to enter all professions who have not, and never can possess, either natural or acquired ability.

The objection has been urged that such a law would be oppressive upon many honorable and well meaning practitioners, who have not enjoyed all the advantages now open to the student, and consequently are deficient in their knowledge of many details. Laws are made for the transgressors of the right. Such a law should and doubtless would be framed and enacted as would be restraining to the dishonest and incompetent; and certainly no one would desire that either the one or the other should perform so responsible and important a duty for himself, neither would he permit it, if he had a proper appreciation of the whole subject.

The greatest benefit resulting from such a law would probably be prospective, operative in its influence more upon those who enter the profession hereafter, than those now in it; and still the present tide of quackery ought to be checked as far as possible.

The work now lies at the door of every honorable practitioner of our profession in the State. And does any one ask, "What can I do?" 1st. By all proper means and opportunities endeavor to create a right public opinion on the subject; and 2d. Circulate and get signatures to petitions for such a law. We will send from this office copies of such petitions, with a copy of the proposed law, to all Dentists in the State within a few days; and we would urge their immediate circulation, and when filled let them be placed in the hands of the Legislators of the respective localities; or if that is impracticable, let them be sent to the office of the DENTAL REGISTER. 3d. Let no Legislator, either Senator or Representative, go up from any part of the State on the 1st of January, without fully understanding the subject in all its bearings; let all questions and objections be as fully answered as possible, that they may be able to act understandingly and promptly.

Now then, this matter rests upon the members of the profession throughout the State, which if well performed, we doubt not all will be obtained that is desired. Then let us all go promptly to work.

